# Comparative evaluation of image registration techniques in functional ultrasound imaging

**DOI:** 10.1162/IMAG.a.47

**Published:** 2025-06-20

**Authors:** Shan Zhong, Kofi Agyeman, Shanze Syed, Richard Tobing, Wooseong Choi, Charles Liu, Darrin Lee, Vassilios Christopoulos

**Affiliations:** Alfred E. Mann Department of Biomedical Engineering, University of Southern California, Los Angeles, CA, United States; Neuroscience Graduate Program, University of California Riverside, Riverside, CA, United States; Department of Bioengineering, University of California Riverside, Riverside, CA, United States; Department of Computer Science and Engineering, University of California Riverside, Riverside, CA, United States; Department of Neurological Surgery, Keck School of Medicine, University of Southern California, Los Angeles, CA, USA; USC Neurorestoration Center, Keck School of Medicine, University of Southern California, Los Angeles, CA, USA; Rancho Los Amigos National Rehabilitation Center, Downey, CA, United States

**Keywords:** functional ultrasound imaging, mouse neuroimaging, image registration, rigid transformation, non-rigid transformation

## Abstract

Functional ultrasound imaging (fUSI) is an emerging hemodynamic-based neuroimaging technique that combines high spatiotemporal resolution and sensitivity with extensive brain coverage, enabling a wide range of applications in preclinical brain research. Based on power Doppler imaging, fUSI measures changes in cerebral blood volume by detecting the back-scattered echoes from red blood cells moving within its field of view. Despite the significant contribution of fUSI technology to neuroscience research, its full potential is partly constrained by the challenge of accurately co-registering power Doppler vascular maps acquired across different sessions and/or animals to a single reference: an approach that could widen the scope of experimental paradigms and enable the utilization of advanced data analysis tools. This study aims to address this critical limitation by comparing eight image registration techniques to align 2D sagittal whole-brain fUSI datasets acquired from 82 anesthetized mice. The results showed a significant improvement in the alignment of fUSI images across all techniques. However, the non-rigid registration methods demonstrated either similar or superior performance in similarity metrics compared to rigid approaches, with the non-rigid version of*Elastix*and*Imregdeform*emerging as the top-performing techniques. Further analysis revealed that both methods maintained comparable high levels of geometric integrity, as evidenced by similar mean Jacobian determinants (close to 1) and low folding rates. In summary, our study offers the first comparative analysis of image registration techniques specifically tailored for 2D fUSI mouse brain datasets, paving the groundwork for enhanced utilization of fUSI in future research applications.

## 1 Introduction

Functional ultrasound imaging (fUSI) represents a rapidly advancing neuroimaging modality for large-scale recordings of neural activity through neurovascular coupling (Bercoff et al., 2011;Mace et al., 2013;Macé et al., 2011;Tanter & Fink, 2014). It is minimally invasive and provides a unique combination of great spatial coverage (12.8 mm width×10.0 mm penetration depth), high spatiotemporal resolution (∼ 100 µm, up to 10 ms), and high sensitivity (∼ 1 mm/s velocity of blood flow). The enhanced spatiotemporal properties allow for measuring the activity of small neuronal populations, providing a closer connection to underlying neuronal signal compared to other hemodynamic methods, such as functional magnetic resonance imaging (fMRI). The first*in vivo*proof-of-concept for fUSI was established in 2011 by imaging the cerebral blood volume (CBV) changes in the micro-vascularization of the rat brain during whisker stimulation (Macé et al., 2011). Since then, fUSI has been applied to image brain activity during olfactory stimuli (Osmanski, Martin, et al., 2014), resting-state functional connectivity (Osmanski, Pezet, et al., 2014), behavioral tasks in freely moving rodents (Sieu et al., 2015), and non-human primates (Dizeux et al., 2019;Norman et al., 2021). While fUSI has been applied across a wide range of studies (Deffieux et al., 2021), most of them are limited to averaging activity within selected regions of interest (ROIs) (Norman et al., 2021;Rabut et al., 2020;Vidal et al., 2020) and/or performing ROI-to-ROI functional connectivity analysis across sessions and animals (de Paz & Macé, 2021;Osmanski, Pezet, et al., 2014). Despite the significant contribution of these studies, they inherently depend on predefined ROIs, which may overlook spatially distributed patterns of brain activity. A more comprehensive alternative would evaluate brain activity at the maximum spatial resolution of fUSI, treating each pixel as a unit for analysis. This pixel-based analysis offers several advantages, including the ability to perform unbiased, whole-brain comparisons and identify localized changes in activity or connectivity without relying on predefined ROIs. Despite its potential, pixel-based analysis has not yet been widely adopted in fUSI studies, primarily because it requires precise co-registration of all recorded power Doppler (pD) images to a common reference. Note that although some ultrasound scanners like the Iconeus One use 3D angiography to map the brain and register fUSI images to the Allen CCFv3 for consistent plane acquisition across animals (Brunner et al., 2020;Nouhoum et al., 2021), substantial variability still exists due to differences in brain size and shape. Therefore, to enable pixel-based analysis and fully leverage the spatial resolution of fUSI, it is critical to develop robust methods for co-registering and aligning pD images across animals to a common reference. This will enable investigators to explore a wider spectrum of experimental paradigms, leverage more advanced machine-learning tools, pixel-based analysis and generate statistical parametric maps (SPMs) from data pooled from multiple sessions and across animals.

In the current study, we optimized and evaluated the effectiveness of eight image registration techniques that have been widely used in computer vision and neuroimaging studies (Avants et al., 2009;Hickey et al., 2023;Jenkinson & Smith, 2001;Jenkinson et al., 2002;A. Klein et al., 2009;Modat et al., 2010,^2014^;Pnevmatikakis & Giovannucci, 2017;Shamonin et al., 2014;Soloukey et al., 2020;Vercauteren et al., 2009). We utilized 2D sagittal whole-brain fUSI data acquired from anesthetized mice and demonstrated that the alignment of registered fUSI images was significantly improved compared to the pre-registration “moving” images (i.e., images requiring spatial transformation to align with a reference image). After parameter optimization and evaluation, our findings showed that non-rigid techniques demonstrated comparable or superior performance to rigid techniques for cross-subject alignment. Among the various registration techniques examined, the non-rigid versions of*Elastix*and*Imregdeform*emerged as the top-performing methods, achieving excellent alignment as indicated by high similarity metrics. Importantly, our additional analysis of geometric properties revealed that both methods maintained comparable levels of geometric integrity, with similar mean Jacobian determinants and low folding rates. This comprehensive assessment highlights the importance of evaluating registration methods across multiple performance dimensions to select techniques appropriate for specific research applications. Overall, the current study offers a detailed comparison of image registration techniques for 2D fUSI brain data, promoting more effective use of fUSI in neuroimaging research settings.

## 2 Methods

### 2.1 Animals

The dataset used in the current study is part of another research project performed by our team recently to understand the mechanism of medial septal nucleus (MSN) deep brain stimulation (DBS) on CBV changes following 0.1 mg/kg MK-801 drug administration (Crown et al., 2024). Eighty-two male 8–12-week-old C57BL/6 mice (Charles River Laboratories; Hollister, CA) were used in this study. They were fed*ad libitum*and maintained at a regular light-dark cycle of 12 h. Mice were anesthetized with 5% isoflurane in${\text{O}}_{2}$ /${\text{N}}_{2}$O (1:2) carrier gas and then maintained at a constant rate (1.5-2%) through the experiment. Body temperature was kept constant throughout fUSI recordings by placing animals on an electric heating pad. The mice were then visually monitored for respiration and reflexes. Hair was removed from the animals’ heads using a commercially available depilatory cream (Nair, Pharmapacks). They were then head-fixed in a stereotaxic frame with ear bars to stabilize their heads. Echographic gel was then applied onto the imaging window. All procedures were approved by the Institutional Animal Care and Use Committee of University of Southern California (IACUC #21006).

### 2.2 Data acquisition

**Fig. 1.**
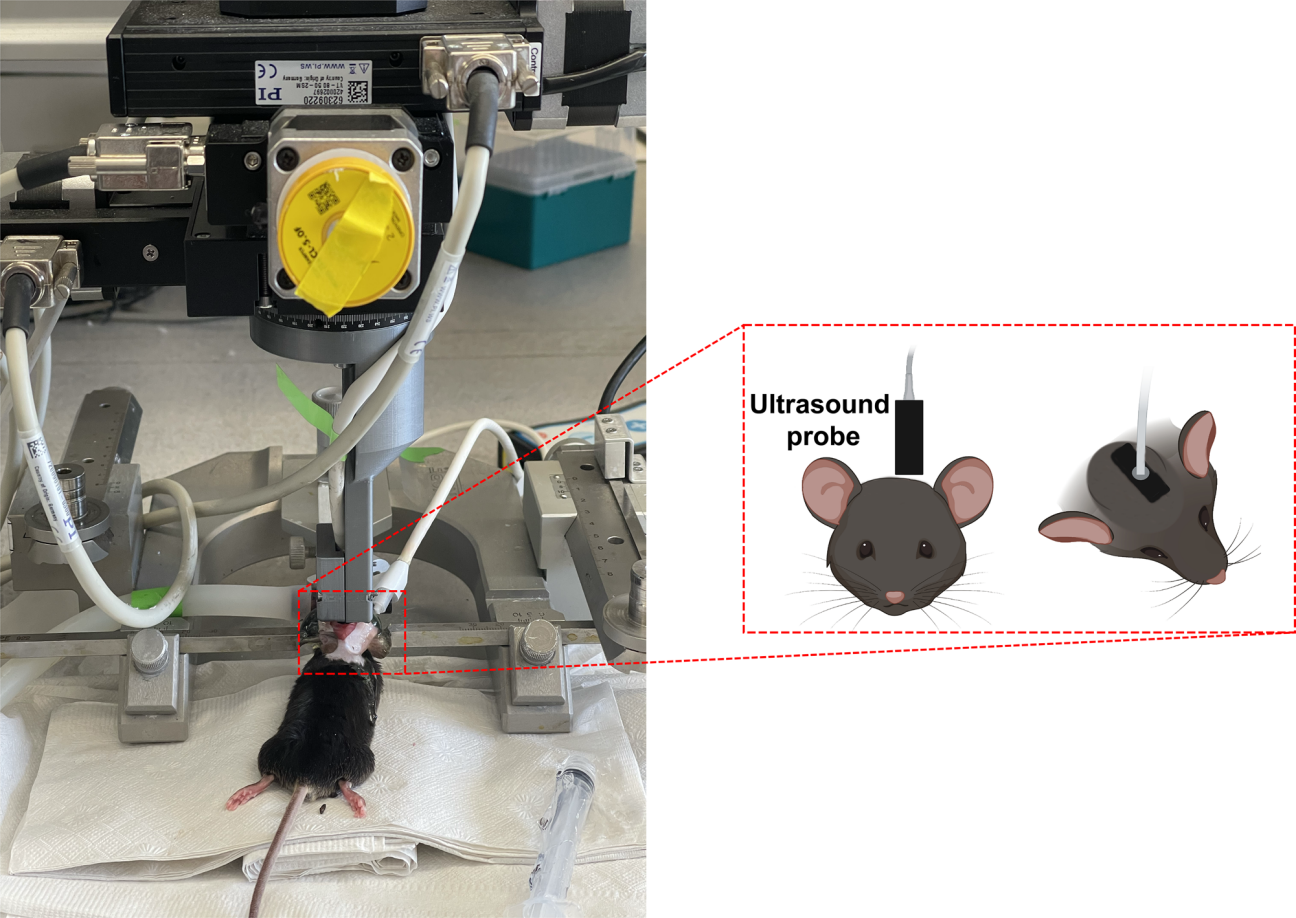
Experimental setup and recordings. An illustrative diagram showing the motorized acquisition system used for 2D fUSI scanning in a mouse brain.

Transcranial ultrafast ultrasound image acquisition was performed using the Iconeus One (Iconeus, Paris, France) scanner. Images were obtained using a 15 MHz linear array probe (128 elements, 0.1 mm pitch), positioned on the intact skull and skin following hair removal and the application of echographic gel. The probe remained steadily fixed on a motorized system throughout the experiment (Fig. 1). Prior to recordings, the target plane was determined by performing a 3D whole-brain fUSI image acquisition for each mouse, and co-registering with a standard Allen Mouse Common Coordinates Framework brain atlas utilizing dedicated software available with the Iconeus system (Q. Wang et al., 2020). pD images were acquired with a spatial resolution of 100 µm × 100 µm, 400 µm slice thickness, and 12.8 mm (width) × 10 mm (depth) field of view (FOV). Each image was obtained from 200 compounded frames acquired with a 500 Hz frame rate, with each frame built using 11 tilted plane waves (from -10° to +10°, increment by 2°) acquired at 5500 Hz pulse repetition frequency. These frames were processed into pD images using coherent compounding within the Iconeus One scanner (Mace et al., 2013). fUSI recording sessions were performed using real-time continuous acquisition, with successive blocks of 400 ms, separated by 600 ms pause, generating a frame rate of 1 Hz. A 5-min baseline recording was obtained from each of the 82 mice, followed by either MK-801 drug or saline injection, after which recording continued for an additional 40 min without manipulation. In the current study, the last 2 min of the baseline recordings were utilized to evaluate the performance of the optimized image registration techniques.

### 2.3 Registration preparation

We selected 13 animals in which their average pD vascular maps show the most details compared to the other animals (seeFig. S1in the Supplementary Materials). These were designated as reference mice. For each reference mouse, we computed the mean of the first ten images to use as the reference image.

### 2.4 Registration techniques

We optimized and evaluated eight image registration techniques. An overview of the methods is shown inTable 1.

**Table 1.** The evaluated image registration techniques.

Name	Platform	Transform	Metric	Optimization method	Number of parameters optimized
NoRMCorre	Linux/Windows/OSX	Rigid/piece-wise rigid (non-rigid)	Normalized cross-correlation	One-pass (optimization methods could be added)	4 (rigid) / 6 (non-rigid)
Imregtform	Linux/Windows/OSX	Rigid	Mutual information (mean squares available)	(1 + 1) evolutionary optimization strategy (gradient descent available)	5
Elastix	Linux/Windows/OSX	Rigid & non-rigid	Normalized cross-correlation (other metrics available)	Adaptive stochastic gradient descent (others available)	3 (rigid) / 4 (non-rigid)
Imregdemons	Linux/Windows/OSX	Non-rigid	Sum of squared difference	Gradient descent	3
Imregdeform	Linux/Windows/OSX	Non-rigid	Mutual information + a regularization term	Gradient-based optimization	3
FLIRT ^a^	Linux/Windows/OSX	Rigid	Correlation ratio	Powell’s optimization	3
NiftyReg	Linux/Windows/OSX	Rigid & non-rigid	Normalized cross-correlation (rigid) Normalized mutual information + bending energy (non-rigid)	Gradient descent (others available)	5
ANTs ^b^	Linux/Windows/OSX	Rigid & non-rigid	Mutual information (other metrics available)	Gradient descent (others available)	4

a FSL FMRIB’s Linear Image Registration Tool.

b Advanced Normalization Tools.

#### 2.4.1 NoRMCorre

*NoRMCorre*has been commonly used in solving large scale image registration problems in functional neuroimaging (Pnevmatikakis & Giovannucci, 2017). It operates by splitting the FOV into overlapping spatial patches (i.e., frames) along all directions. The patches are registered at a sub-pixel resolution for rigid translation against a regularly updated template. To enable a fair comparison between different registration techniques, we deactivated the template updating function, ensuring that the same reference image was consistently used as the template for each round of registration. The estimated alignments are subsequently up-sampled to create a smooth motion field for each image that can efficiently approximate non-rigid artifacts in a piecewise-rigid manner. Note that the*NoRMCorre*technique could operate in both rigid and non-rigid (piecewise-rigid) fashion, both of which were deployed in our study.

#### 2.4.2 Elastix

The*Elastix*toolbox is a modular software package widely used for intensity-based image registration of medical images (S. Klein et al., 2009). It applies a transformation matrix to pixels of pre-registration moving images, mapping them onto a reference image, forming a coordinate map for each pixel. In practice,*Elastix*loops over all pixels in the reference image, computes the mapped position, interpolates the moving image at the new location, and fills these values into the resulting post-registration moving image. It utilizes various key components, such as transformation model, sampler, metric, optimizer, interpolator, and image masks. All these components are adjustable. In this study, we opted to use the Euler transformation (a rigid transformation) and B-spline transformation (a non-rigid transformation) as our transformation models. To measure the transformation efficiency, we initially utilized a sampler strategy to select locations (i.e., pixels) in the reference image for the metric to evaluate. While using all pixels from the reference image is the most straightforward but time-consuming approach, alternatives such as “Random” and “Random Coordinate” sampling strategies are also available. The latter samples not only pixel positions but also positions between pixels.

The next step is to select a similarity metric that measures the degree of similarity between the reference and the moving images. Among different metrics, we selected the advanced normalized correlation, which provides a criterion that is optimized using the Adaptive Stochastic Gradient Descent (ASGD) method that has good convergence properties and is relatively fast in combination with the random sampling strategy. ASGD estimates the optimal transform parameters by enabling the transformation matrix to be adjusted in each iteration. This is accomplished by taking adaptive step sizes toward the direction that minimizes the normalized mutual information (NMI) function (i.e., cost functionC) as shown inEq. 1.

$$\mu_{k+1}=\mu_{k}-a_{k}g(\mu_{k})$$ (1)

where$g(\mu_{k})=\frac{\partial C}{\partial \mu }$represents the gradient of the cost functionCwith respect to the transformation parameterμevaluated at the current position$\mu_{k}$, and$a_{k}$is the gain factor. Moreover, a transformed point is generally mapped to a non-grid position, which is not precisely aligned with the exact grid position of a pixel. During optimization, the transformed point requires interpolation to evaluate the image intensity. B-spline interpolation was executed using a cubic B-spline function. This function calculates a weighted average based on the surrounding pixels. The weight assigned to each surrounding pixel is determined by its distance to the point where the interpolated value is needed.

#### 2.4.3 Imregtform

We utilized*Imregtform*, a function native to the Image Processing Toolbox in Matlab that performs intensity-based image registration. This technique operates on the principle of identifying an optimal geometric transformation to align the moving image to a reference image (MathWorks, 2023a). To achieve this, a (1 + 1) evolutionary optimization strategy that draws inspiration from the basic principles of natural selection and mutation was implemented (Goldberg, 1989). This approach is a simple yet effective form of evolutionary algorithm used for optimization problems. In this method, a single parent produces one single offspring in each generation through mutation. If the offspring’s fitness is better or equal to the parent’s, it replaces the parent in the next generation (Beyer & Schwefel, 2002). This process continues iteratively until a satisfactory solution is found.

The*Imregtform*function allows the use of various transformation types to adjust the spatial relations of the images under consideration. These include translation (shift in X and Y coordinates), rigid (translation plus rotation), affine (includes scaling and shearing), and similarity transformations (preserves shape, includes rotation, translation, and scaling) (MathWorks, 2023a). In this study, we specifically implemented rigid transformation for the registration process.

#### 2.4.4 Imregdemons

We also utilized*Imregdemons*, a function that is available in the Image Processing Toolbox in Matlab, to implement a variation of the “Demons” algorithm proposed byThirion (1996,^1998^). This technique adopts the concept of diffusing models to perform image-to-image matching. The major principle of the Demons algorithm lies in its approach to estimate a displacement field that maps the moving image onto the reference image. It works under the assumption that the intensity of a given point should be the same in both the moving and reference images, otherwise a “force” or “demons” is created that pushes the moving image towards the reference image. This idea is similar to how Maxwell’s approach resolved the Gibbs paradox in thermodynamics. The exerted forces were conceptualized from the principles of optical flow equations (Barron et al., 1994), and are proportional to the intensity difference and the gradient of the reference image.

The displacement resulting from the application of the force by the demons is given by the equation:

$$d=\frac{\left(m-s\right)\nabla s}{{\left(\nabla s\right)}^{2}+{\left(m-s\right)}^{2}}$$ (2)

wheredis the displacement during one iteration step,mandsare the intensities of the moving and reference images, respectively, and∇sis the gradient in each nodal point of the reference image (Thirion, 1998). The algorithm alternates between computing these forces and performing regularization through elementary Gaussian smoothing. The Demons algorithm was then further refined to enforce diffeomorphic transformations (Vercauteren et al., 2009), which is implemented by the*Imregdemons*function.

#### 2.4.5 Imregdeform

*Imregdeform*is part of the Matlab Image Processing Toolbox (since 2022b) (MathWorks, 2023b). It utilizes a parametric approach for image registration with total variation regularization. This approach is characterized by the incorporation of an isotropic total variation (Christiansen et al., 2007) and an efficient local correlation coefficient (LCC), which is defined as the combined weighted correlation coefficients of image intensity levels, computed over n-dimensional patches centered at each pixel. The isotropic total variation is defined by the following equation (Vishnevskiy et al., 2016):

$$E_{R}^{TV}(d)=\upsilon \sum_{l\leq L}\sqrt{\sum_{i,j\leq N}{\left(\nabla_{i}d_{j}\left[l\right]\right)}^{2}}=\upsilon {\left\|D(d)\right\|}_{2,1}$$ (3)

wheredis the displacement field,$\nabla_{i}d_{j}[l]$denotes the spatial gradient of thej-th displacement component at locationl, andυis the pixel volume. The term${\left\|D(d)\right\|}_{2,1}$is the2,1-norm of the displacement gradient matrixD(d). The displacement gradient matrixD(d)is defined as (Vishnevskiy et al., 2016):

$$D(d)={\left[\nabla_{1}d_{1}\nabla_{2}d_{1}\ldots \nabla_{1}d_{2}\ldots \nabla_{N}d_{N}\right]}^{T}\in {\mathbb{R}}^{N^{2}\times L}$$ (4)

with each term representing the spatial gradient for each component of the displacement field in each dimension. This method provides an efficient solution scheme via the alternating directions method of multipliers (ADMM), enabling better convergence in practice than conventional comparable methods.

#### 2.4.6 FSL FLIRT

FSL*FLIRT*is a robust linear registration tool widely used in neuroimaging research (Jenkinson & Smith, 2001;Smith et al., 2004). The tool employs the correlation ratio (CR) as its primary cost function. For two images A and B, the CR is computed as:

$$\eta (B|A)=1-\frac{1}{N\sigma^{2}}\sum_{i}N_{i}\sigma_{i}^{2}$$ (5)

whereNis the total number of pixels in the overlapping region between images,$\sigma^{2}$is the total variance of imageB,$N_{i}$is the number of pixels in thei-th iso-intensity set of imageA(pixels having the same intensityi), and$\sigma_{i}^{2}$is the variance ofBintensities for pixels corresponding to thei-th iso-intensity set ofA.

For optimization,*FLIRT*employs a multiresolution strategy combined with a one-dimensional search method. This method optimizes each transformation parameter (e.g., translation, rotation) individually while keeping other parameters fixed, cycling through all parameters iteratively until convergence. This approach balances computational efficiency and registration accuracy, making it well-suited for linear image registration tasks.

#### 2.4.7 NiftyReg

*NiftyReg*is a flexible registration toolbox supporting both rigid and non-rigid transformations, widely used in medical imaging research. Rigid registration is performed using a block-matching strategy that divides images into small blocks and identifies correspondences using normalized cross-correlation (NCC). Given any two images${\text{I}}_{\text{a}}$and${\text{I}}_{\text{b}}$, the NCC could be computed as:

$$NCC({\text{I}}_{\text{a}},{\text{I}}_{\text{b}})=\frac{\sum ({\text{I}}_{\text{a}}(k)-\mu_{{\text{I}}_{\text{a}}})({\text{I}}_{\text{b}}(k)-\mu_{{\text{I}}_{\text{b}}})}{\sigma_{{\text{I}}_{\text{a}}}\sigma_{{\text{I}}_{\text{b}}}}$$ (6)

wherekdescribes the pixels within the images,$\mu_{{\text{I}}_{\text{a}}}$and$\mu_{{\text{I}}_{\text{b}}}$represent the mean intensities of images${\text{I}}_{\text{a}}$and${\text{I}}_{\text{b}}$, respectively, and$\sigma_{{\text{I}}_{\text{a}}}$and$\sigma_{{\text{I}}_{\text{b}}}$represent the standard deviations of the intensities in${\text{I}}_{\text{a}}$and${\text{I}}_{\text{b}}$.

Transformation parameters are then estimated through an iterative Trimmed Least Squares (TLS) approach (Ourselin et al., 2001). To enhance robustness and computational efficiency,*NiftyReg*also employs a multiresolution approach, beginning with coarse alignment and progressively refining at higher resolutions (Modat et al., 2014).

For non-rigid registration,*NiftyReg*uses a Free-Form Deformation (FFD) model based on cubic B-splines (Rueckert et al., 1999). Registration is guided by an objective function combining NMI for multimodal similarity and a bending energy penalty term to ensure plausible deformations (Modat et al., 2010). Optimization is performed via gradient descent.

#### 2.4.8 ANTs

*ANTs*is a state-of-the-art framework designed to handle complex image registration tasks with precision and versatility (Avants et al., 2009). It supports both rigid and non-rigid registration methods, providing robust solutions for a variety of alignment challenges. In particular, non-rigid registration in*ANTs*is powered by the symmetric normalization (SyN) algorithm, which computes smooth and invertible deformations to align local anatomical features (Avants et al., 2008). SyN optimizes a time-varying velocity field while incorporating regularization terms to preserve anatomical topology. Metrics such as cross-correlation or mutual information guide the alignment, making SyN particularly effective for capturing fine-grained structural variations. In this study, rigid and non-rigid registration were evaluated separately to assess their individual performance.

#### 2.4.9 Testing combined rigid and non-rigid registration

To further investigate the potential benefits of combining methods, we implemented a two-step registration strategy for non-rigid techniques that lacked built-in initial alignment functionality. Specifically, we first applied the optimized*Imregtform*function to achieve rigid alignment. The output of this rigid registration step was then used as the input for each optimized non-rigid method.

This two-step approach ensured consistent initial alignment across all non-rigid techniques, allowing for a fair comparison of their performance. While some toolboxes, such as*ANTs*, offer integrated pipelines that perform both initial alignment and non-rigid registration in a single step, this was not available across all methods. By standardizing the initial alignment process, we mitigated potential variability and ensured that differences in performance could be attributed to the non-rigid component.

### 2.5 Optimization of image registration techniques using genetic algorithms (GAs)

**Fig. 2.**
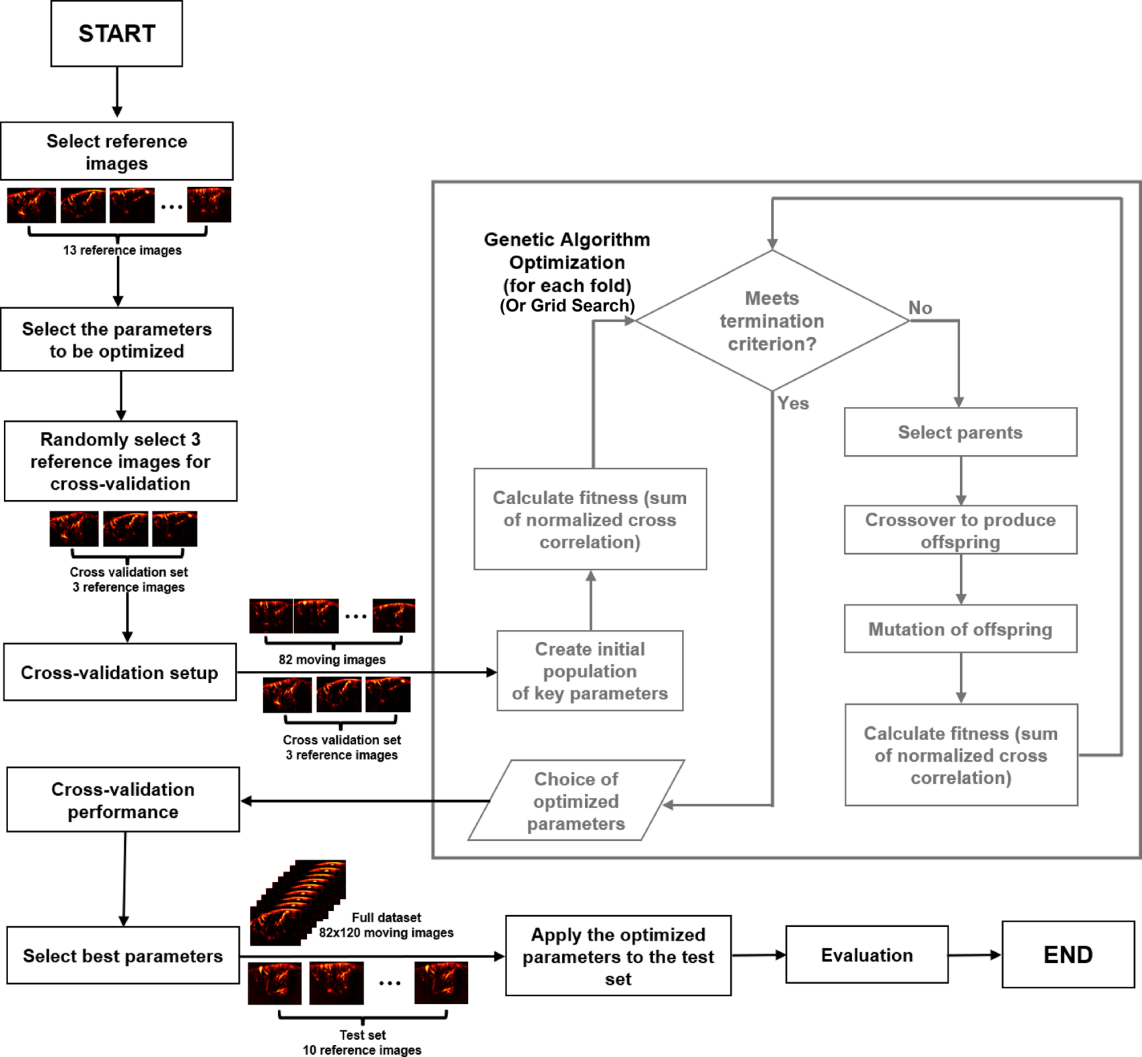
Flow chart of the optimization pipeline.

To enhance the performance and ensure a fair comparison of the image registration techniques, we employed a genetic algorithm (GA) for optimizing their key parameters (Fig. 2). We selected 13 animals and computed the mean of the first ten images of each animal to serve as reference images, that is, 13 references in total. During the GA, we registered the first image from each of the 82 animals (pre-registration moving image) against the selected reference image. Our approach involved a cross-validation technique to achieve a robust and generalizable outcome. We split the 13 reference images into a training set of 3 randomly selected images, and a test set of 10 reference images. This approach was intended to mitigate the computation demands of the GA. The GA was then applied to the training set utilizing a cross-validation approach across three iterations. In each iteration, a distinct combination of reference images was utilized, that is, two for training, and one for validation. This rotational method guaranteed that each image was selected as a validation set once, facilitating a thorough evaluation of the optimization across diverse data instances.

In order to balance between computational demands and the quality of registration results, we carefully selected the parameters for optimization based on preliminary analyses and existing knowledge, focusing on those with a substantial influence on registration accuracy (for detailed parameters utilized and those selected for optimization, refer to theSupplementary Materialssection). We optimized these key parameters by employing the “Matlab’s GA toolbox”. We selected NCC as the fitness function in the optimization process due to its robustness to variations in brightness and contrast across images. The fitness function is designed to measure the overall quality of alignment by aggregating the NCC values for each post-registration moving image relative to its corresponding reference image. As the GA aims to minimize the fitness function, we negated the sum of NCC values, directing the optimization towards enhancing image alignment. The fitness function is calculated as:

$$\text{Fitness}=-\sum_{j=1}^{\text{N}}\sum_{i=1}^{\text{n}}NCC\left({\text{I}}_{{\text{image}}_{i}},{\text{I}}_{{\text{ref}}_{j}}\right)$$ (7)

where$I_{{\text{image}}_{i}}$denotes thei-th image in the experimental dataset consisting ofnimages, and$I_{{\text{ref}}_{j}}$represents thej-th reference image out of a total ofNreference images. Note thatN=2, since we registered the moving images to two reference images and then aggregated the NCC values. Additionally,n=82denotes the total number of moving images in GA optimization procedure, that is, each moving image corresponds to the first image out of 2 min of baseline recordings in each animal.

We set the maximum number of generations to 100 times the number of variables, a function tolerance of 1e-6, and a maximum stall generation limit of 50, where the algorithm stops if the average relative change in the best fitness function value of 50 generations (specified by MaxStallGenerations) is less than or equal to the function tolerance (specified by FunctionTolerance). In other words, the optimization procedure is completed if there is no significant improvement in the fitness value over 50 consecutive generations, indicating convergence to a solution.

Note that we did not optimize the*Elastix*rigid and non-rigid registration techniques using GA due to their extensive computation time. The computational intensity of*Elastix*can be attributed to the large number of parameters, the multi-resolution registration approach and its workflow, which involves the creation and deletion of temporary directories for each registration task. To overcome this limitation, we selected a few key parameters within the recommended range and employed a grid search optimization process (seeSupplementary Materialsfor these parameters). This approach involved testing a representative set of parameter combinations spanning the defined range through the same cross-validation procedure, enabling the identification of an optimal parameter set without the computational burden of GA. The same approach was applied to the rigid and non-rigid methods of*ANTs*, the rigid and non-rigid methods of*NiftyReg*, and FSL*FLIRT*.

Finally, we selected the optimization parameters that achieved the best cross-validated fitness function, and assessed the performance of the registration methods on a test set consisting of the 10 unused reference images. For testing, we utilized the entire 2-min baseline recordings from each of the 82 mice, yielding a total of 9840 images. We independently registered this dataset to each of the 10 reference images and assessed the performance of the registration techniques using various metrics as detailed in the following section.

### 2.6 Evaluation measures

We employed a range of metrics to assess the efficacy of the image registration techniques. These included NCC, which was used as fitness function in the GA optimization process, along with the multi-scale structural similarity index measure (MS-SSIM), the Haar wavelet-based perceptual similarity index (HaarPSI), dice similarity coefficient (DSC), and Jacobian determinant.

#### 2.6.1 Normalized cross-correlation

We computed the 2-dimensional NCC between the moving images (before and after registration) and the reference images, considering spatial shift between the two images and measuring their similarity at different relative positions. This process generates a 2-dimensional NCC matrix, where each element represents the NCC value for a specific spatial shift. The highest value in this matrix represents the peak correlation achieved, while the width of this peak reflects the robustness of the correlation. A narrower peak implies more accurate alignment between the compared elements. This approach was also applied to an ROI of cropped images.

#### 2.6.2 MS-SSIM

The MS-SSIM is a similarity measure that extends the classic SSIM by evaluating structural similarity across multiple image resolutions (Z. Wang et al., 2003). This approach involves applying successive low-pass filters and downsampling the input images iteratively, enabling a comprehensive analysis of structural similarity at varying scales. The method computes luminance, contrast, and structural similarity components at different scales, combining them into a single measure using the following equation:

$$\text{MS}-\text{SSIM}({\text{I}}_{\text{a}},{\text{I}}_{\text{b}})={\left[l_{M}({\text{I}}_{\text{a}},{\text{I}}_{\text{b}})\right]}^{\alpha_{M}}\prod_{j=1}^{M}{\left[c_{j}({\text{I}}_{\text{a}},{\text{I}}_{\text{b}})\right]}^{\beta_{j}}{\left[s_{j}({\text{I}}_{\text{a}},{\text{I}}_{\text{b}})\right]}^{\gamma_{j}}$$ (8)

where$l_{M}(x,y)$,$c_{j}(x,y)$, and$s_{j}(x,y)$are the luminance, contrast, and structure measures at scalej, withMbeing the total number of scales. The exponents$\alpha_{M}$,$\beta_{j}$, and$\gamma_{j}$assign different weights to the components, reflecting their relative importance. This design allows MS-SSIM to provide a detailed and robust measure of image similarity. We computed the MS-SSIM using the Matlab multissim function.

#### 2.6.3 HaarPSI

The HaarPSI evaluates the perceptual similarity of two greyscale images,${\text{I}}_{\text{a}}$and${\text{I}}_{\text{b}}$, by combining local and global similarity information derived from Haar wavelet coefficients (Reisenhofer et al., 2018). The local Haar similarity (HS) is computed for each pixel using horizontal and vertical Haar wavelet filters$g_{k}$(k=1, 2), as follows:

$$HS_{{\text{I}}_{\text{a}},{\text{I}}_{\text{b}}}^{\left(k\right)}[x]=l_{\alpha }\left(\frac{1}{2}\sum_{j=1}^{2}S\left(\left|g_{j}^{\left(k\right)}*{\text{I}}_{\text{a}}\left[x\right]\left|,\right|g_{j}^{\left(k\right)}*{\text{I}}_{\text{b}}[x]\right|,C\right)\right)$$ (9)

where*denotes convolution,Cis a small constant for numerical stability, and$l_{\alpha }$is a logistic function enhancing perceptual relevance. The global HaarPSI score aggregates the local similarities, weighted by low-frequency Haar wavelet coefficientsW(p), and could be computed as follows:

$${\text{HaarPSI}}_{{\text{I}}_{\text{a}},{\text{I}}_{\text{b}}}=l_{\alpha }^{-1}{\left(\frac{\sum_{x}\sum_{k=1}^{2}HS_{{\text{I}}_{\text{a}},{\text{I}}_{\text{b}}}^{\left(k\right)}[x]\cdot W_{{\text{I}}_{\text{a}},{\text{I}}_{\text{b}}}^{\left(k\right)}[x]}{\sum_{x}\sum_{k=1}^{2}W_{{\text{I}}_{\text{a}},{\text{I}}_{\text{b}}}^{\left(k\right)}[x]}\right)}^{2}$$ (10)

where$HS_{p}$is the local similarity at pixelp, andW(p)represents the weight derived from the low-frequency wavelet coefficients at that pixel. For more details seeReisenhofer et al. (2018).

#### 2.6.4 Dice similarity coefficient

DSC compares the similarity between the moving images and reference images and is defined as follows:

$$DSC=\frac{2\left|{\text{I}}_{1}\cap {\text{I}}_{2}\right|}{\left|{\text{I}}_{1}\left|+\right|{\text{I}}_{2}\right|}$$ (11)

where${\text{I}}_{1}$and${\text{I}}_{2}$represent two binary images,∩represents the intersection operation, and|⋅|represents the size of a set. In other words, DSC is defined as twice the area of overlap between two images divided by the total number of pixels in both images. Before computing the DSC, we use theimbinarizefunction in Matlab to binarize the 2-dimensional surfaces by thresholding using Otsu’s method (Otsu, 1975). This approach selects a global threshold that minimizes the inter-class variance of the thresholded black and white pixels. Then, we compute the DSC between the moving images (pre- and post- registration) and each of the reference images.

#### 2.6.5 Jacobian determinant analysis

To quantitatively assess the geometric distortion introduced by non-rigid registration techniques, we computed the Jacobian determinant of the deformation fields, measuring local area changes during the transformation.

For a 2D deformation field

$$\vec{\phi }(x,y)=(\phi_{x}(x,y),\phi_{y}(x,y))$$

that maps a point from the moving image to the fixed image, the Jacobian matrixJ(x,y)at each pixel(x,y)is defined as:

$$J(x,y)=\left(\begin{array}{cc} \frac{\partial \phi_{x}}{\partial x} & \frac{\partial \phi_{x}}{\partial y}\\ \frac{\partial \phi_{y}}{\partial x} & \frac{\partial \phi_{y}}{\partial y} \end{array}\right).$$ (12)

The Jacobian determinant|J(x,y)|is computed as the determinant of this matrix. A value of 1.0 indicates no local area change, values greater than 1.0 indicate local expansion, and values less than 1.0 indicate local contraction. Negative Jacobian determinant values indicate topology-breaking folding in the deformation field.

To quantify the overall geometric distortion introduced by each registration technique, we computed the mean Jacobian determinant across all pixels:

$$\text{Mean Jacobian}=\frac{1}{N}\sum_{i=1}^{N}J_{i},$$ (13)

whereNis the total number of pixels and$J_{i}$is the Jacobian determinant at thei-th pixel. We performed this analysis for*Imregdeform*, the non-rigid version of*Elastix*, and*Imregdemons*.

### 2.7 Statistical analysis

#### 2.7.1 Statistical parametric mapping (SPM)

Statistical Parametric Mapping (SPM) is a widely used statistical technique in functional neuroimaging for analyzing signal changes, such as pD signals, induced by specific interventions. In this study, we utilized SPM to evaluate the regional hemodynamic changes in both registered and unregistered pD images following MK-801 administration. For the registered dataset, optimized*imregdeform*was applied prior to SPM analysis to align the images. We evaluated the effects of MK-801 by comparing pD signal changes between baseline activity (defined as the 2 min prior to injection) and the final 2-min before stopping the recordings. The baseline corresponds to frames 181–300 (2 min prior to injection), while the final 2-min period corresponds to frames 2581–2700 (38–40 min post-injection).*p*-Values were computed for each pixel by comparing pD signals between the baseline and the final 2 min of recordings post-injection, with multiple comparisons corrected using the false discovery rate (FDR). To prevent overcrowding in the SPM maps, we retained the top 30%of pixels exhibiting significant changes (FDR-corrected*p*<0.001). For visualization, we calculated and displayed z-scores of percent pD signal change from baseline for these top 30%of significant pixels.

#### 2.7.2 ANOVA and post-hoc analysis

In addition to SPM, statistical analyses were performed using one-way ANOVA followed by Tukey–Kramer post hoc tests for multiple comparisons. All analyses were conducted using Matlab R2022b.

## 3 Results

### 3.1 Visualization of fUSI image registration outcomes

We assessed eight distinct image registration techniques as outlined inTable 1, using fUSI recordings from 82 anesthetized mice. Note that we evaluated both the rigid and non-rigid transformations of the*NoRMCorre*,*Elastix*,*NiftyReg*, and*ANTs*methods. Additionally, for non-rigid techniques, we implemented a combined two-step registration approach. This approach involved first applying the optimized rigid transformation using*Imregtform*, followed by the corresponding non-rigid transformation. We selected 13 animals with high-quality vascular maps to serve as references (seeFig. S1in the Supplementary Materials). We then computed the mean vascular map from the first 10 acquired images from each of these mice to generate the reference images. Analysis of frame-to-frame stability in these reference mice demonstrated exceptionally high temporal consistency (mean NCC values>0.9998, seeFig. S4in the Supplementary Materials).

We further split the 13 reference images into a cross-validation set of 3 images and a test set of 10 images, and optimized the key parameters of these techniques using GA or grid search approach (for details seeSection 2). After optimization, we utilized a dataset comprising of 9840 moving images (2-min recordings from 82 mice) to evaluate the registration techniques on the test set.

**Fig. 3.**
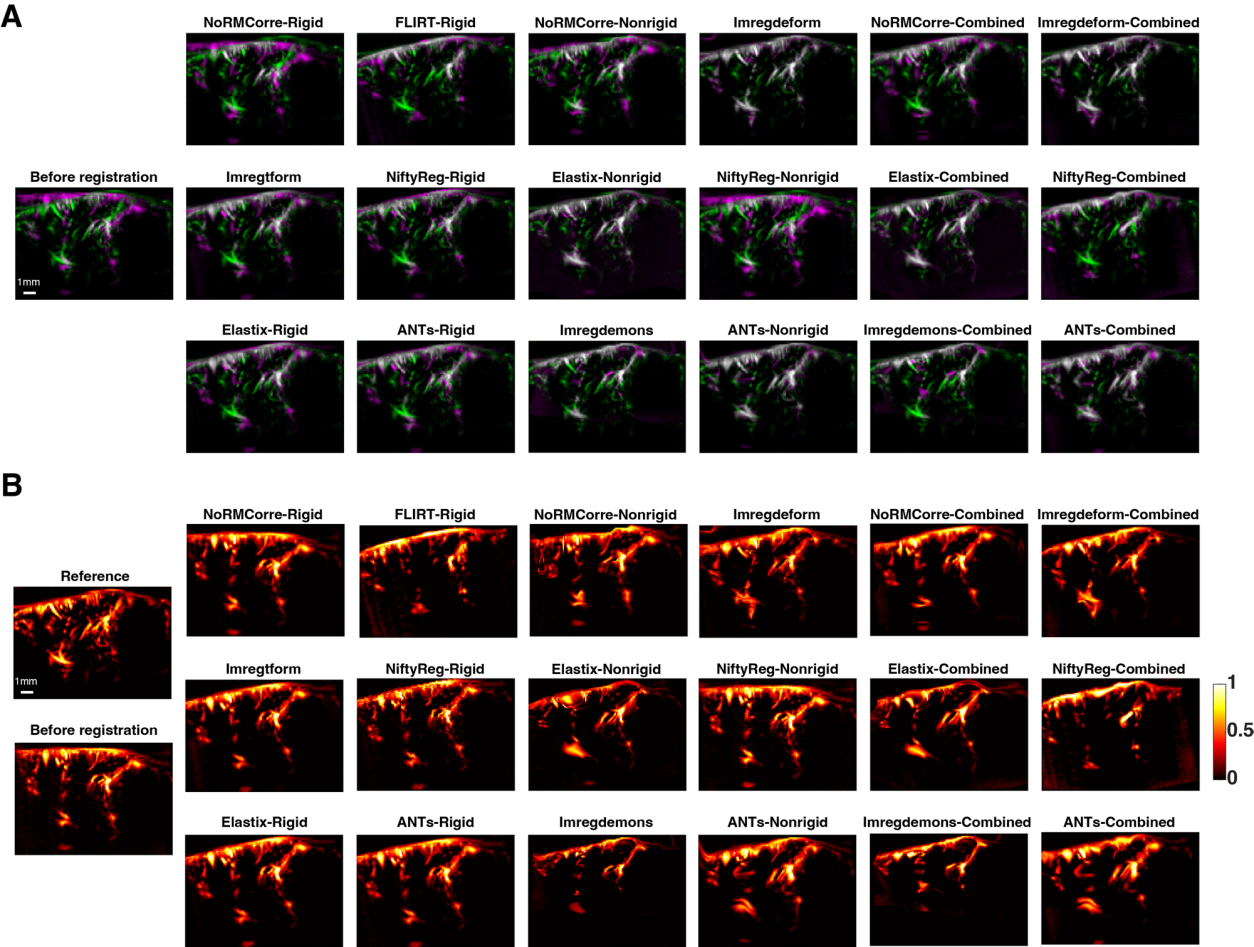
Visualization of fUSI image registration outcomes. (A) Representative examples of image registration between the vascular maps of two different mice. The single left panel illustrates the initial misalignment before registration, while the remaining panels display the registered results with overlay visualization. Each column represents a different registration technique, categorized as rigid, non-rigid, or combined approaches. Green: reference image. Magenta: moving image (pre-registration in the left single panel, post-registration in the other panels). (B) Same with panel (A) but without overlay of the reference in the pre-registration (left single panel) and post-registration (remaining panels) images. The color bar represents normalized intensity values (0 to 1).

Figure 3Apresents the registration outcomes using an overlay visualization method. The single left panel represents the initial misalignment between the average vascular maps of two example mice before registration, with the reference image shown in green and the moving image shown in magenta. The remaining panels display the overlaid vascular maps after registration using different techniques. Visual comparison shows enhanced alignment between the fixed and moving images for all techniques compared to the pre-registration state, as evidenced by increased overlap (white regions) of vascular structures.Figure 3Bpresents the intensity distributions of the vascular structures before and after registration, without overlaying the reference image, enabling a direct assessment of potential registration-induced distortions. Most of the non-rigid techniques seem to result in better alignment compared to the rigid ones, with the exception of the non-rigid version of*NiftyReg*. However, some of the non-rigid techniques (for instance, the non-rigid version of*NoRMCorre*and*Imregdemons*) result in notable distortion artifacts. Among the combined methods,*NoRMCorre-Combined*exhibited improved preservation of anatomical structures compared to its non-rigid counterpart. The combined version of*NiftyReg*, although showing better alignment than its non-rigid version, generated more pronounced artifacts in the registered images.

**Fig. 4.**
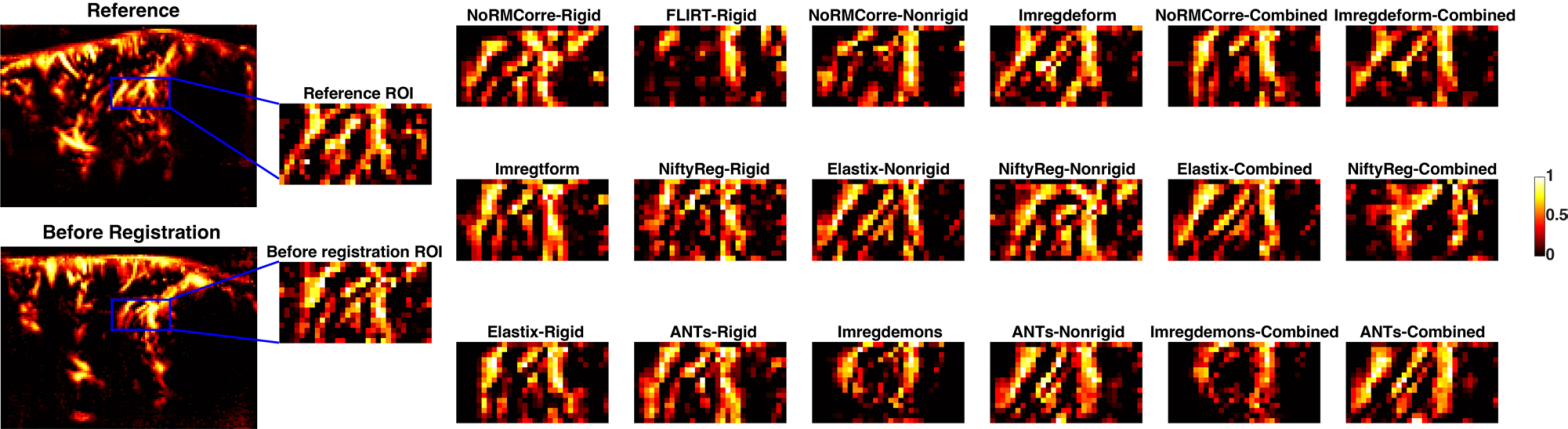
Registration outcomes in an ROI. The left panels display the reference image (top) and an example moving image before registration (bottom), with zoomed-in views of a selected ROI (blue boxes). The zoomed-in ROIs on the right show the registered results for each evaluated technique. The color bar represents normalized intensity values (0 to 1).

To provide a closer examination of registration performance within a localized area (smaller vessels),Figure 4illustrates the registration outcomes in a selected local ROI for all evaluated techniques. While most rigid and non-rigid techniques improved alignment compared to the pre-registration state,*FLIRT-Rigid*performed poorly, failing to match the reference image effectively. In contrast,*Imregdemons*introduced large distortions within the ROI. Among the non-rigid methods,*Imregdeform*, the non-rigid version of*Elastix*, and the non-rigid version of*ANTs*achieved good alignment of small vessels within the ROI.

### 3.2 Quantitative evaluation of registration techniques

**Fig. 5.**
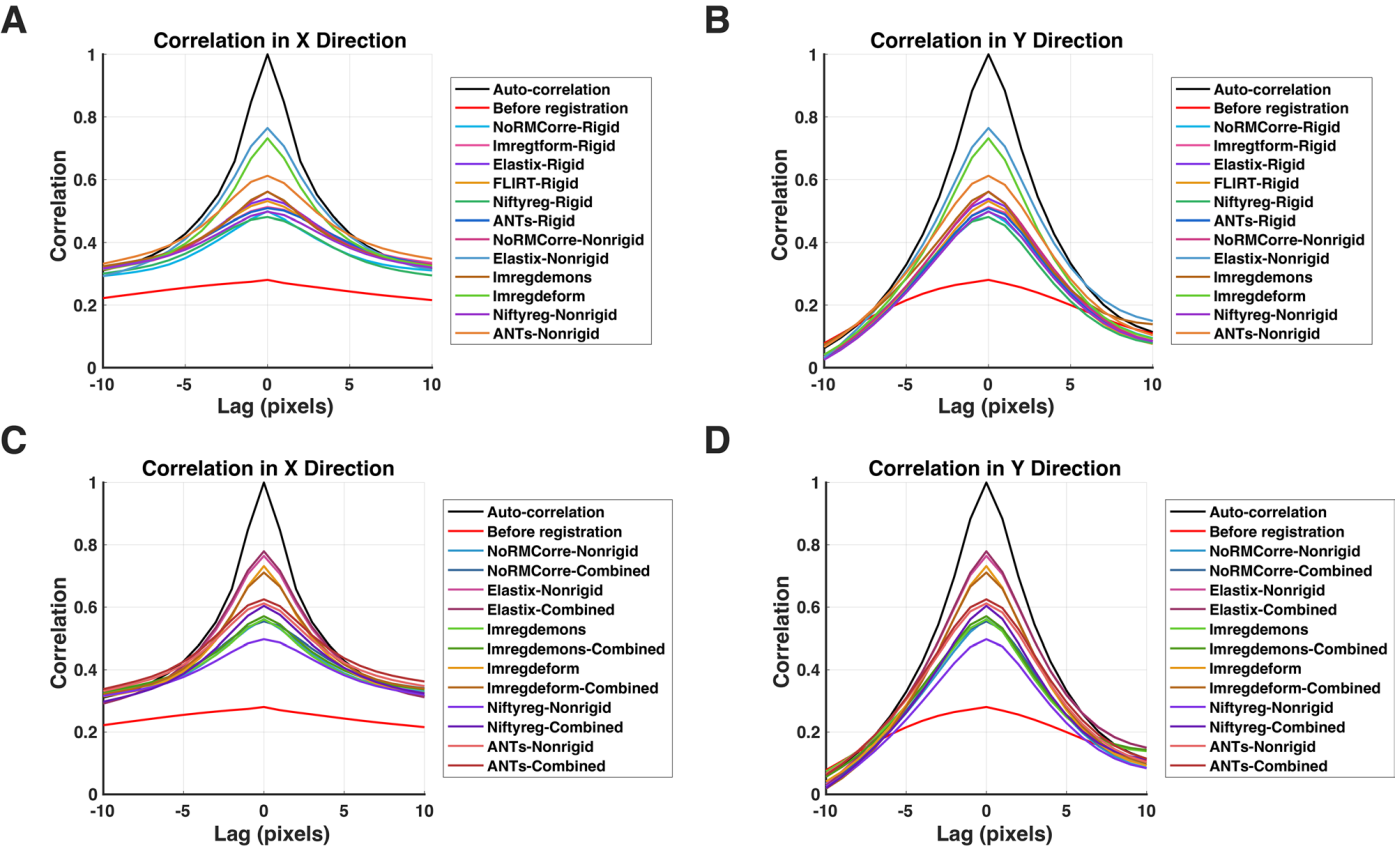
NCC analysis between the moving images and the reference images. (A) The mean NCC plot between the moving images (pre- and post-registration) and the reference images in the x direction across 10 reference images. Reference image auto-correlation is also shown (black curve). (B) Same as (A) but for the y direction. (C) and (D) display additional comparisons of NCC for the x and y directions, respectively, including results for pre-registration moving images, reference image auto-correlation, and all non-rigid and combined registration techniques.

To quantify the performance of the image registration techniques, we first registered the dataset of 9840 moving images to each of the 10 reference images in the test set, generating 10 groups of post-registration moving images for each registration technique. We then calculated the 2-dimensional NCC between each reference image and its corresponding moving and registered images, averaging the NCC values across the 10 reference images in both the x and y directions. As shown inFigure 5A(x direction) andFigure 5B(y direction), the NCC between the reference and the post-registration moving images shows a stronger (value range from 0.48 to 0.78) and sharper (width at half maximum range from 1.10 to 3.00 mm for x direction, and 0.60 to 0.90 mm for y direction) peak located in the center, compared to the NCC between the reference and the pre-registration moving images both in x and y directions (value = 0.28, width at half maximum = 3.00 mm for x direction and 1.40 mm for y direction). As a reference, the NCC between the reference image with itself (i.e., autocorrelation) yields a width at half maximum = 1.00 mm for x direction and 0.40 mm for y direction. The non-rigid version of*Elastix*exhibits the highest peak in comparison to all other non-rigid and rigid techniques, closely followed by the non-rigid technique*imregdeform*, and then the non-rigid version of*ANTs*. The remaining techniques showed similar NCC peaks and widths.Figure 5C(x direction) andFigure 5D(y direction) provide an additional comparison for all non-rigid and combined registration techniques. All combined methods exhibited performance comparable to their respective non-rigid techniques. Notably, the combined*NiftyReg*method outperformed the non-rigid*NiftyReg*, which showed poor performance. Overall, applying any registration technique improved NCC compared to the pre-registration baseline (red, lowest trace).

We conducted a similar analysis on the selected ROI (shown inFig. 4) to evaluate the registration performance in a localized region (seeFig. S2). The results were consistent with the global analysis shown inFigure 5, demonstrating similar trends across registration techniques. However, due to the smaller frame size, the NCC peaks were less symmetrical compared to the global analysis.

**Fig. 6.**
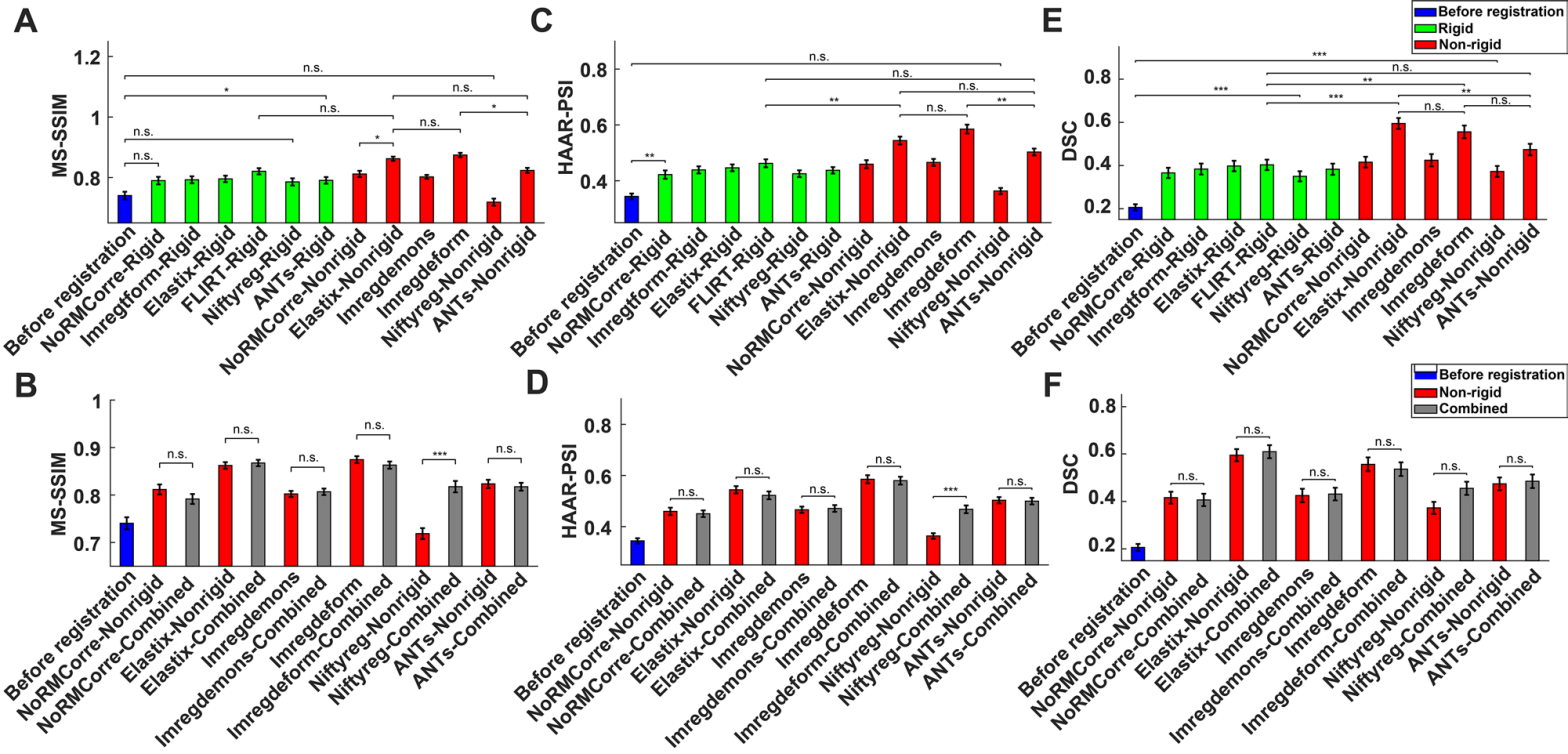
Quantitative evaluation of image registration techniques using MS-SSIM, HaarPSI, and DSC. (A) MS-SSIM for the moving images before registration, after rigid registration, and after non-rigid registration across 10 reference images; (B) MS-SSIM for the moving images before registration, after non-rigid registration, and after the corresponding combined registration techniques. (C) HaarPSI following the same registration sequences as in panel (A). (D) HaarPSI following the same registration sequences as in panel (B). (E) DSC following the same registration sequences as in panel (A). (F) DSC following the same registration sequences as in panel (B).

To further evaluate the performance of the image registration techniques, we computed the mean MS-SSIM values for each slice within the moving images (pre- and post-registration) relative to the reference image across the 10 reference images. MS-SSIM computes structural similarity across multiple resolutions, quantifying the preservation of structural details in the registered images. Higher MS-SSIM values indicate better preservation of structural details and more accurate alignment. The MS-SSIM values were analyzed across different registration approaches, including pre-registration moving images, rigid registration techniques (e.g., the rigid versions of*NoRMCorre*and*NiftyReg*), and non-rigid registration techniques (e.g.,*Imregdeform*, the non-rigid versions of*Elastix*and*ANTs*) (Fig. 6A). Statistical analysis using a one-way ANOVA revealed significant differences among the groups (p<0.001). Tukey’s post-hoc analysis showed no significant improvement in MS-SSIM for the rigid version of*NoRMCorre*(p=0.0517), the rigid version of*NiftyReg*(the lowest-performing rigid technique,p=0.1125), or the non-rigid version of*NiftyReg*(the lowest-performing non-rigid technique,p=0.9573) compared to the pre-registration baseline. In contrast, all other techniques showed significant improvements (p<0.05). Among the non-rigid techniques,*Imregdeform*achieved the highest MS-SSIM values, though it was not significantly different from the non-rigid version of*Elastix*(p=0.9998). The non-rigid version of*Elastix*and the non-rigid version of*ANTs*demonstrated comparable performance (p=0.2966), though*Imregdeform*showed significantly better performance compared to the non-rigid version of*ANTs*(p=0.0375).Figure 6Bpresents the MS-SSIM values for the pre-registration moving images, the non-rigid techniques, and their corresponding combined registration techniques (e.g., the combined version of*NiftyReg*, the combined version of*Elastix*). A one-way ANOVA revealed significant differences among these groups (p<0.001). Tukey’s post-hoc analysis showed that the combined techniques performed similarly to their corresponding non-rigid techniques (p>0.05). Notably, the combined version of*NiftyReg*significantly outperformed the non-rigid version of*NiftyReg*(p<0.001).

In addition, we computed the mean HaarPSI values for each slice within the moving images (pre- and post-registration) relative to the reference image across the 10 reference images. HaarPSI is a perceptual similarity metric based on Haar wavelet coefficients, quantifying the alignment quality by assessing local and global intensity similarities between the moving and reference images. Higher HaarPSI values indicate better perceptual similarity and more visually consistent alignment of the moving images to the reference images. The HaarPSI values were analyzed across different registration approaches, including the pre-registration moving images, the rigid registration techniques, and the non-rigid registration techniques (Fig. 6C). Statistical analysis using a one-way ANOVA revealed significant differences among the groups (p<0.001). Tukey’s post-hoc analysis indicated a significant improvement in HaarPSI for all techniques compared to the pre-registration baseline (p<0.01), except for the non-rigid version of*NiftyReg*(the lowest-performing non-rigid technique,p=0.9979). Among the non-rigid methods,*Imregdeform*achieved the highest HaarPSI values and significantly outperformed the non-rigid version of*ANTs*(p<0.01). The non-rigid version of*Elastix*(second best) demonstrated comparable performance to*Imregdeform*(p=0.5763) and the non-rigid version of*ANTs*(p=0.5805). The non-rigid version of*ANTs*(third best) performed similarly to the FSL*FLIRT*(p=0.6033). Both*Imregdeform*and the non-rigid version of*Elastix*significantly outperformed FSL*FLIRT*(p<0.01).Figure 6Dpresents the HaarPSI values for the pre-registration moving images, the non-rigid techniques, and their corresponding combined registration techniques. A one-way ANOVA revealed significant differences among these groups (p<0.001). Tukey’s post-hoc analysis showed that the combined methods exhibited similar performance to their corresponding non-rigid techniques (p>0.05), except for the combined version of*NiftyReg*, which significantly outperformed the non-rigid version of*NiftyReg*(p<0.001).

Furthermore, we computed the mean of DSC, a widely accepted metric for assessing the similarity (i.e., overlap) between two sets (seeSection 2for more details), across the 10 reference images. The DSC ranges from 0 (no overlap) to 1 (perfect overlap). Similar to the other two metrics, we first compared the DSC values for the pre-registration moving images, the rigid registration techniques, and the non-rigid registration techniques (Fig. 6E). A one-way ANOVA revealed significant differences among the groups (p<0.001). Tukey’s post-hoc analysis indicated significant improvement in DSC for all techniques compared to the pre-registration baseline (*p*<0.001), including the lowest-performing rigid technique, the rigid version of*NiftyReg*(*p*<0.001). Among the rigid techniques, FSL*FLIRT*achieved the highest DSC values but showed no significant difference compared to the non-rigid version of*ANTs*(*p*= 0.7315). FSL*FLIRT*, however, was significantly outperformed by*Imregdeform*(second highest,p<0.01). Among the non-rigid techniques, the non-rigid version of*Elastix*achieved the highest DSC values and showed no significant difference compared to*Imregdeform*(p=0.9967). Similarly, no significant difference was observed between*Imregdeform*and the non-rigid version of*ANTs*(p=0.5036), though the non-rigid version of*Elastix*significantly outperformed the non-rigid version of*ANTs*(p<0.05).Figure 6Fshows the DSC values for the pre-registration moving images, the non-rigid techniques, and their corresponding combined registration techniques. A one-way ANOVA revealed significant differences among these groups (p<0.001). However, Tukey’s post-hoc analysis showed no significant differences between the combined techniques and their corresponding non-rigid methods (p>0.05). Specifically, the combined version of*NiftyReg*performed similarly to the non-rigid version of*NiftyReg*(p=0.5899).

**Fig. 7.**
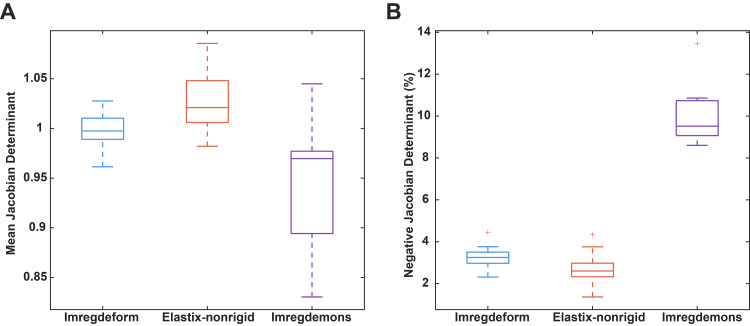
Comparison of geometric distortion across three non-rigid registration techniques. (A) Boxplot showing the mean Jacobian determinant. (B) Boxplot showing the percentage of pixels with negative Jacobian determinants (folding rate). Data are based on registration of the entire dataset to 10 reference images.

To further assess the registration quality of the best-performing techniques based on similarity metrics, we evaluated the geometric distortion introduced by two top-performing non-rigid registration techniques,*Imregdeform*and the non-rigid version of*Elastix*, and included*Imregdemons*for comparison, as its registered images inFigure 3Bdisplayed noticeably higher distortion level. Geometric distortion was quantified by analyzing the Jacobian determinant of the deformation fields (visualized inFig. S3), where values close to 1 indicate minimal local expansion or contraction, values less than 1 indicate local contraction, values greater than 1 indicate local expansion, and negative values reflect folding artifacts. For each method, the entire dataset was registered to 10 reference images, and we computed the mean Jacobian determinant and the folding rate (i.e., the percentage of pixels with negative Jacobian determinants) across these references. The overall mean Jacobian determinants were1.00±0.63for*Imregdeform*,0.95±1.10for*Imregdemons*, and1.03±0.68for the non-rigid version of*Elastix*, while the corresponding folding rates were3.29%±1.29%,9.98%±0.57%, and2.71%±1.86%, respectively. One-way ANOVA revealed significant differences among the methods for the mean Jacobian determinant (Fig. 7A,p<0.001) and for folding (Fig. 7B,p<0.001). Tukey’s post-hoc analysis indicated that*Imregdemons*produced a significantly lower mean Jacobian determinant than both the non-rigid version of*Elastix*(p<0.001) and*Imregdeform*(p<0.05), and that the folding rate of*Imregdemons*was significantly higher than those of both*Imregdeform*and the non-rigid version of*Elastix*(p<0.001). These findings show that*Imregdemons*exhibits more pronounced overall deformation as well as a significantly higher folding rate, indicating more severe local distortions. In contrast, the similar mean Jacobian determinants and low folding rates of*Imregdeform*and the non-rigid version of*Elastix*demonstrate that these two methods perform comparably well in terms of preserving geometric integrity.

### 3.3 Validation of image registration using SPM analysis to assess CBV changes post-MK-801 administration

To validate our registration approach in a real-world context, we conducted SPM analysis to evaluate the effects of MK-801 administration on CBV changes. Separate analyses were performed using both unregistered and registered pD images from the animals. Registration was performed using*Imregdeform*, which achieved high performance in similarity metrics. By comparing the pD signal between the last 2 min of baseline and the final 2 min of recordings (38–40 min post-MK-801 administration), we identified pixels where the hemodynamic signal significantly changed, indicating alterations in CBV following MK-801 administration. The results presented inFigure 8demonstrate the benefits of registration. In unregistered data (right panel), the activation patterns appeared scattered and biologically implausible, with apparent activity extending beyond brain boundaries—likely artifacts from structural misalignment between frames. In contrast, images processed with*Imregdeform*(left panel) revealed coherent, anatomically-precise activation patterns, primarily within prefrontal cortex and hippocampus. Note that these results are consistent with recent findings showing that MK-801 administration causes strong CBV reduction in hippocampus and prefrontal cortex (Crown et al., 2024). Overall, the difference in the SPM maps between the unregistered and the registered pD images highlights how proper registration enables more accurate functional mapping by removing artifacts caused by structural variability.

**Fig. 8.**
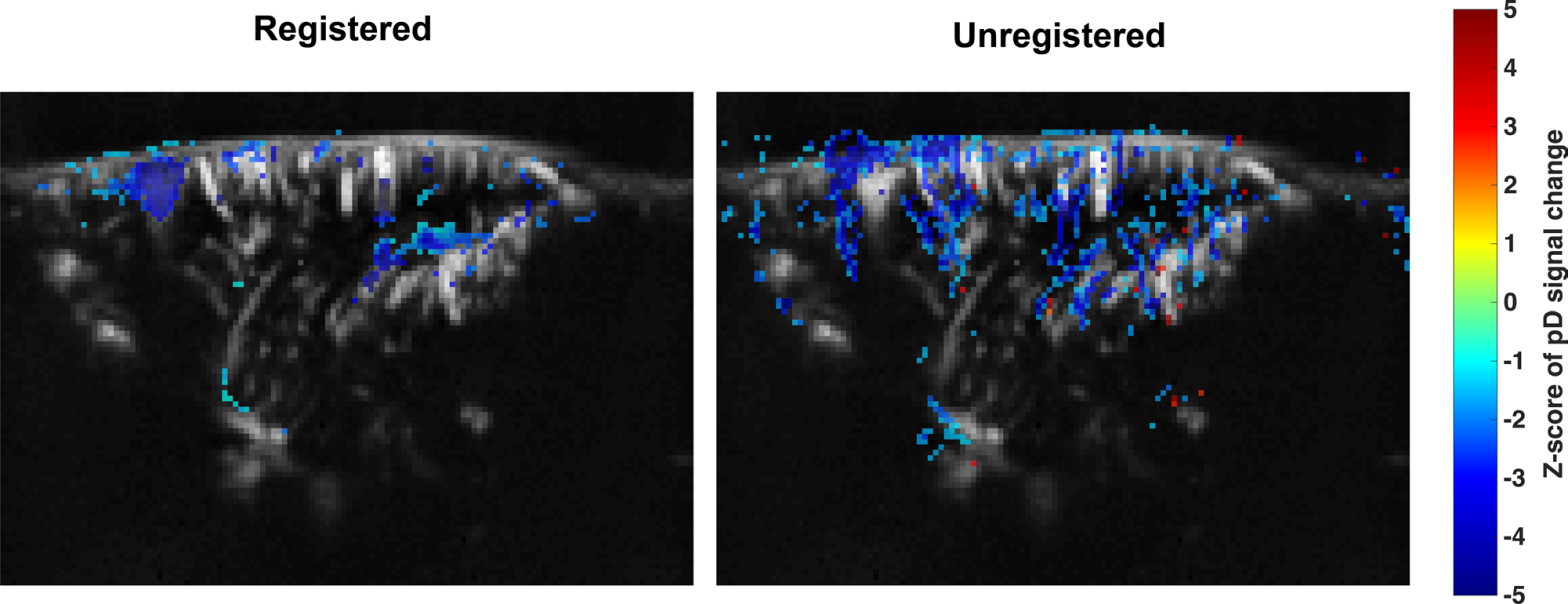
Effect of image registration on SPM analysis of MK-801-induced CBV changes. SPM maps show brain regions with significantly different pD signal between baseline (last 2 min pre-injection) and post MK-801 administration (38–40 min post-injection). The left panel shows results from pD images registered using the optimized*Imregdeform*technique, while the right panel presents unregistered moving images for comparison. The color bar represents z-scores of%pD signal change from baseline in pixels identified as significant.

## 4 Discussion

Image registration represents a critical component in the analysis of neuroimaging data, enabling the integration and comparison of images across subjects and time points. Our comprehensive evaluation of eight image registration techniques for fUSI data alignment has revealed several key insights about their relative performance and applicability. The results demonstrate that non-rigid registration techniques generally exhibited similar or higher evaluation metrics compared to rigid ones, emphasizing the importance of flexibility in registration processes when aligning datasets across different animals. The quantitative analysis in this study showed that the non-rigid implementations of*Elastix*and*Imregdeform*achieved superior alignment according to multiple similarity metrics. Our additional examination of geometric properties revealed that both methods show similar mean Jacobian determinants and low folding rates. The successful implementation of registration, as demonstrated through SPM analysis of MK-801-induced effects using*Imregdeform*, enables more reliable detection of functional changes across subjects. This advancement addresses a crucial limitation in current fUSI studies, which often rely solely on ROI-based analyses, and opens new possibilities for investigating brain function at both global and local scales.

### 4.1 Registration performance in vascular structures

A critical aspect of fUSI registration involves balancing the alignment of large vessels, which serve as primary landmarks due to their strong pD signal, with the preservation of smaller cortical vessels that often carry functionally relevant information. Our optimization of registration parameters through genetic algorithms and grid search methods revealed important insights about this balance. The key challenge lies in finding parameter configurations that allow sufficient flexibility for accurate alignment while preventing artifactual deformations, particularly in regions containing smaller vessels.

In non-rigid registration techniques, regularization parameters proved essential for achieving this balance. For example, in*Imregdeform*, the grid regularization parameter weights the regularization term in the optimization process (Vishnevskiy et al., 2016). Higher values produce smoother displacement fields that maintain structural integrity but may under-register small vessels, while lower values permit more localized deformations necessary for precise vessel alignment but risk introducing anatomically implausible distortions. Similar trade-offs were observed in other non-rigid techniques, such as*Elastix*, where grid spacing parameters required careful optimization to balance global alignment with local structural preservation.

### 4.2 Limitations and future perspectives

While our study represents a significant step forward in fUSI image registration and data analysis, several aspects require consideration for future development. Our evaluation approach utilized multiple independent metrics, enabling objective assessment of registration performance. This comprehensive evaluation strategy provided robust validation of image registration techniques across our experimental conditions. However, despite our systematic optimization using GA and grid search methods, the optimized parameters may not be universally optimal for all fUSI applications. Several factors contribute to this limitation, including variations in vascular architecture across subjects, differences in imaging protocols and hardware configurations between laboratories, and the inherent trade-offs between registration accuracy and computational efficiency. The dynamic nature of functional imaging data and potential variations in signal-to-noise ratio suggests that researchers may need to fine-tune these parameters for their specific experimental contexts. This challenge of parameter optimization across diverse experimental settings highlights a broader need in the field for standardized methodological frameworks. Beyond parameter optimization, a fundamental challenge in non-rigid registration is balancing alignment accuracy with anatomical preservation. While our Jacobian analysis quantifies geometric distortion, without a universally accepted brain vascular atlas, establishing a true gold standard for evaluating registration fidelity remains difficult. This limitation means that techniques achieving high similarity metrics may still introduce subtle anatomical distortions that could impact biological interpretations. The current absence of a standardized fUSI vascular atlas in the field presents both a limitation and an opportunity. Future development of standardized validation approaches and reference atlases specifically designed for fUSI data would further strengthen systematic evaluations of registration methods while providing better measures for assessing the biological validity of registered images.

From an experimental design perspective, the exclusive use of male mice in our study, while helping to control hormonal variability in our drug response evaluation (Crown et al., 2024), represents a limitation in understanding potential sex-specific differences in vascular architecture and response patterns. Future studies should extend these registration approaches to both male and female animals to ensure broader applicability. Similarly, while the use of isoflurane anesthesia provided stable imaging conditions essential for this methodological study, the extension of these registration techniques to awake and freely moving animals presents additional challenges that warrant further investigation. In awake settings, motion artifacts introduce complex distortions that may require more sophisticated registration techniques. These motion-related challenges are particularly pronounced in freely moving animals, where significant out-of-plane movements and rotations can occur. Despite these challenges, the demonstrated success of fUSI in awake animals (Dizeux et al., 2019;Norman et al., 2021;Rabut et al., 2019;Sieu et al., 2015;Urban et al., 2015) suggests promising opportunities for extending these registration techniques to more naturalistic settings. Future work should address how the performance of these registration methods might be affected by motion-related variability and explore complementary approaches, such as motion tracking systems, to maintain registration accuracy in less constrained experimental conditions.

From a technical perspective, our implementation focused on several practical choices that could be expanded in future work. The use of 2D sagittal imaging offered advantages for assessing whole-brain responses and demonstrated the effectiveness of our registration approach in this widely used configuration. However, it has limitations in capturing the full three-dimensional complexity of brain vasculature and function. The observed variability in sagittal plane alignment points to the benefits of extending these methods to 3D registration. In addition, while our use of pD images generated robust results, future studies could investigate whether dB-scaling might further enhance registration performance. The potential integration with multi-modal imaging data could further enhance registration accuracy while providing additional anatomical context.

### 4.3 Conclusion

Our evaluation of eight fUSI registration techniques revealed that non-rigid methods generally achieved similar or superior similarity metric performance compared to rigid approaches. Among the top-performing techniques, the non-rigid versions of*Elastix*and*Imregdeform*excelled in similarity metrics. Further analysis of geometric distortion revealed that both methods maintained geometric integrity, as indicated by comparable mean Jacobian determinants (close to 1) and low folding rates. These findings identify the non-rigid versions of*Elastix*and*Imregdeform*as promising methods for fUSI registration, offering effective performance in terms of both alignment accuracy and geometric preservation. The real-world application of our optimized registration approach was demonstrated through successful implementation of group-level SPM analysis. While we utilized the Iconeus BPS system for co-registration with the Allen Brain Atlas, the registration methods evaluated in our study provide valuable solutions for the entire fUSI community. This advancement in registration methodology provides a foundation for more advanced analyses of fUSI data while establishing a framework for future methodological developments in cross-subject functional brain mapping.

## Supplementary Material

Supplementary Material

## Data Availability

Data will be made available upon a reasonable request and after signing a formal data-sharing agreement.
