# Supplementary material for: Comparative evaluation of image registration techniques in functional ultrasound imaging
Source: Imaging Neurosci (Camb). 2025 Jun 20;3:IMAG.a.47. doi: 10.1162/IMAG.a.47 (PMC12319969; doi:10.1162/IMAG.a.47)
Supplement: Supplementary Material [file imag.a.47_supp.pdf]

Supplementary Materials

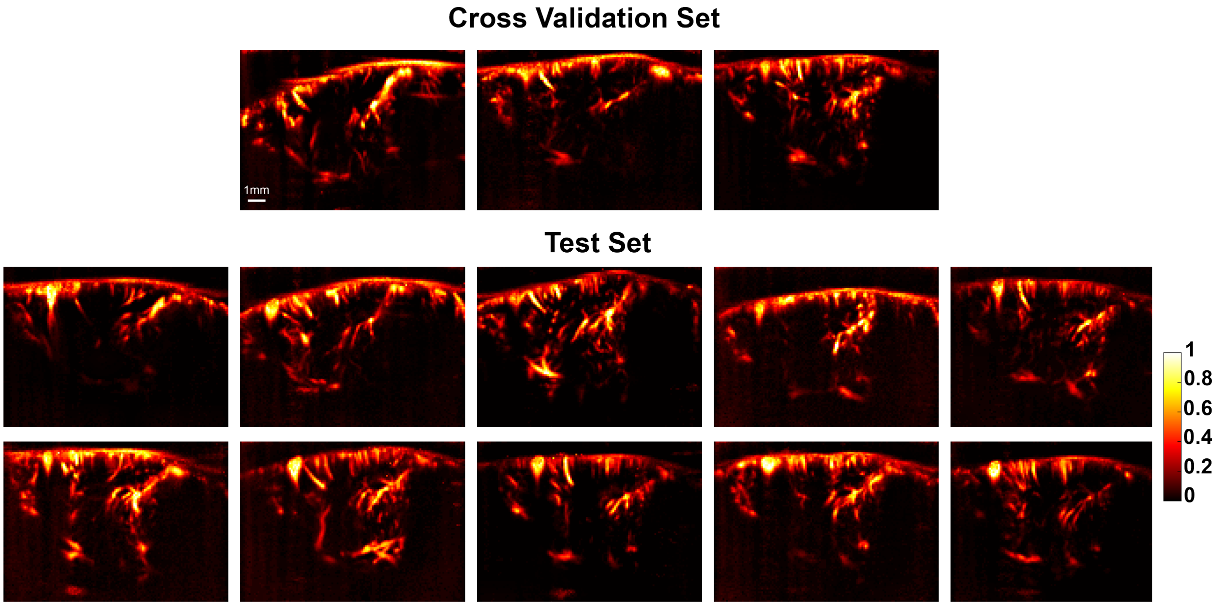

Figure S1. Sagittal power Doppler vascular maps of mouse brains used as reference images in this study. These images represent sagittal planes from 13 different mice, including 3 images used for cross-validation (top row) and 10 images used for testing (middle and bottom rows). Field of view: 12.8 mm (width) × 10 mm (depth). Scale bar: 1 mm.

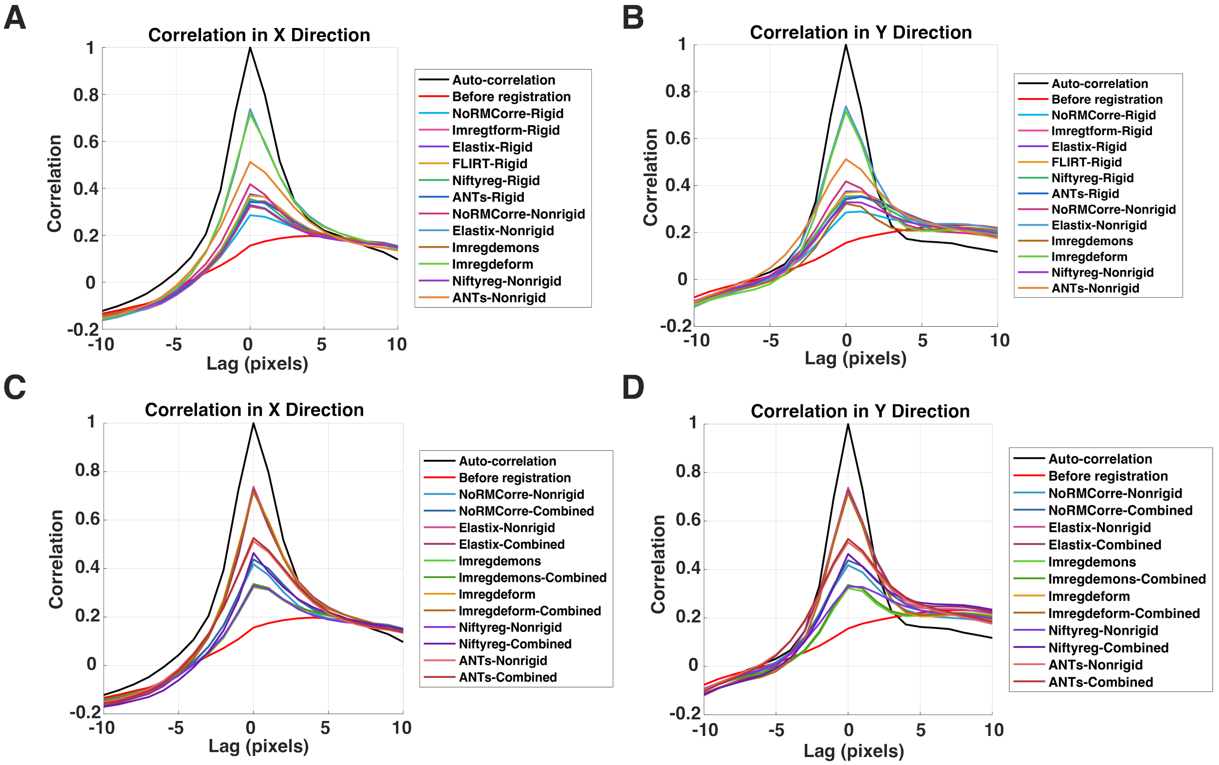

Figure S2. Normalized cross-correlation (NCC) analysis in selected region of interest (ROI) in Fig. 4. (A) The mean NCC plot for the ROI between the moving images (pre- and post-registration) and the reference images in the x direction across 10 reference images. Reference image auto-correlation is also shown. (B) Same as (A) but for the y direction. (C) and (D) display additional comparisons of NCC peaks for the ROI in the x and y directions, respectively, including results for pre-registration moving images, reference image auto-correlation, and all non-rigid and combined registration techniques.

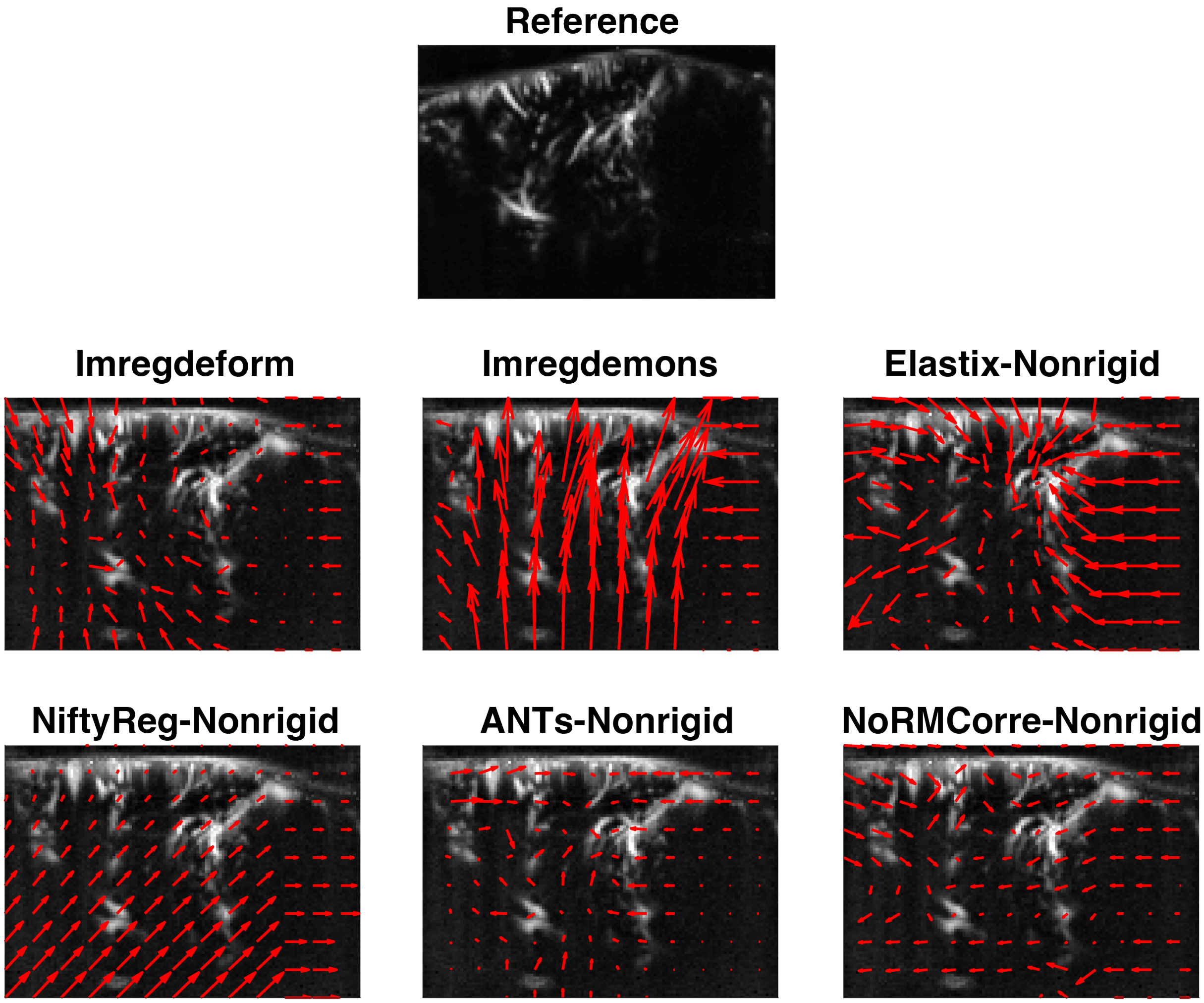

Figure S3. Visualization of displacement vectors in non-rigid registration techniques. Top row shows the reference image. Middle and bottom rows present the displacement vectors from six different non-rigid registration methods overlaid on the moving image. Red arrows indicate the direction and magnitude of local displacements needed to align the moving image with the reference image, with arrow length proportional to displacement magnitude.

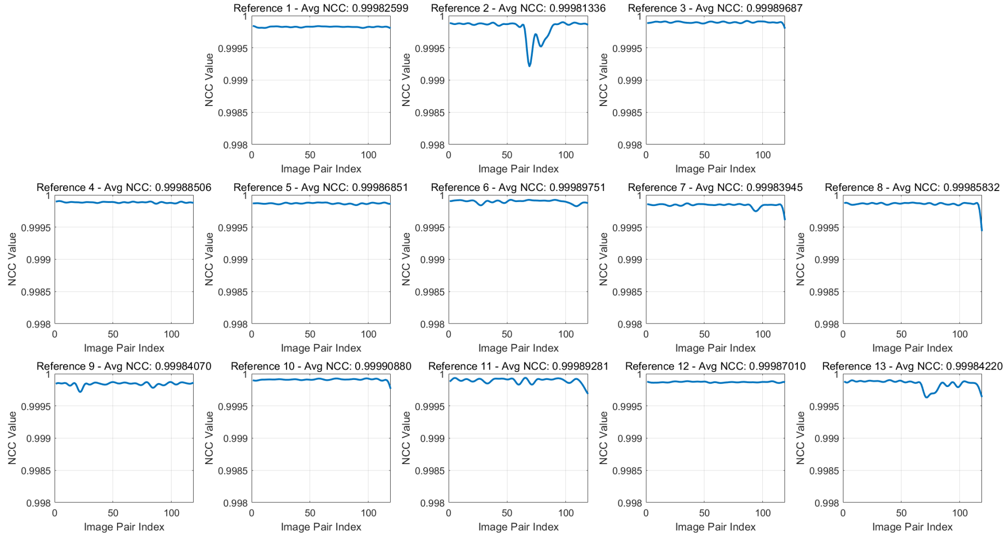

Figure S4. Frame-to-frame stability analysis of reference mice recordings. NCC values between consecutive frames for 2-minute recordings (119 image pairs) from the 13 reference mice. Each subplot displays the frame-to-frame NCC values along the y-axis versus image pair index (1-119) along the x-axis. The first row shows results from the three mice used for optimization, while the remaining subplots show results from the ten mice used for final registration evaluation.

Parameters used for registration techniques

The tables below present the final parameters chosen for the evaluation of all five image registration techniques. Parameters that were optimized via genetic algorithm or via manual tuning, wherein key parameters were chosen from recommended ranges and exhaustively evaluated through systematic combination testing, are highlighted in red.

**Imregtform parameters**		
Parameters	Description	Value
optimizer		registration.optimizer.OnePlusOneEvolutionary
tformType	Type of transform allowed	rigid
optimizer.InitialRadius	Scaling factor for search radius	6.6238e-4
optimizer.epsilon	Convergence threshold	1.302e-4
optimizer.GrowthFactor	Starting step size for search	1.1417
optimizer.MaximumIterations	Max limit for optimization rounds	224
metric		registration.metric.MattesMutualInformation
metric.NumberOfSpatialSamples	Random points for mutual information	500
metric.NumberOfHistogramBins	Bins for histogram computation	50
metric.UseAllPixels	Flag to use all image pixels	1
PyramidLevels	Number of multi-level image pyramid levels	3

NoRMCorre parameters (rigid)		
Parameter	Description	Value
upd_template	flag for online template updating	0
boundary	Method of boundary treatment	copy
us_fac	Upsampling factor for subpixel registration	13
max_shift	maximum rigid shift in each direction	22
phase_flag	flag for using phase correlation	false
shifts_method	Method to apply shifts	linear

NoRMCorre parameters (nonrigid)		
Parameter	Description	Value
upd_template	flag for online template updating	0
boundary	Method of boundary treatment	copy
us_fac	Upsampling factor for subpixel registration	29
max_shift	maximum rigid shift in each direction	13
phase_flag	flag for using phase correlation	false
grid_size	size of non-overlapping regions	[4,4]
overlap_pre	size of overlapping region	[32,32]
min_patch_size	Minimum size of patch	[2,2]
min_diff	Minimum difference between the patches	[16,16]
mot_uf	Degrees of patches upsampling	4
max_dev	Maximum deviation of patch shift from rigid shift	9
shifts_method	Method to apply shifts	linear

Imregdemons parameters		
Parameter	Description	Value
N	Number of interations	129
AccumulatedFieldSmoothing	Smoothing applied at each iteration	0.6212

PyramidLevels	Number of multi-resolution image pyramid levels to use	4

**Imregdeform parameters**		
Parameter	Description	Value
GridSpacing	Grid spacing	[3 3]
PixelResolution	Pixel size	[1 1]
NumPyramidLevels	Number of multi-resolution image pyramid levels to use	6
GridRegularization	Weighing factor for grid displacement regularization	0.2108

**Elastix Parameters (rigid)**		
Parameter	Description	Value
Registration	Class of registration framework	MultiResolutionRegistration
FixedImagePyramid	Morphological scale space	FixedSmoothingImagePyramid
MovingImagePyramid	Morphological scale space	MovingSmoothingImagePyramid
Interpolator	Evaluation of voxel intensity	BSplineInterpolator
Metric	Similarity measure	AdvancedNormalizedCorrelation
Optimizer	Optimal transformation parameter	AdaptiveStochasticGradientDescent
AGSDParameterEstimationMethod	Method for estimating the parameters for Adaptive Stochastic Gradient Descent	DisplacementDistribution
AutomaticTransformInitialization	Automatically guess an initial translation by aligning the geometric centers of the fixed and moving	true

AutomaticScalesEstimation	Method to estimate scales	true
NumberOfSpatialSamples	Samples used to compute similiarity	5000
NewSamplesEveryIteration	Whether to refresh the spatial samples	true
ResampleInterpolator	Intensity interpolation during resampling	FinalBSplineInterpolator
Resampler	Calculation of deformed image	DefaultResampler
Transform	Deformation used	EulerTransform
AutomaticScalesEstimation	Scales between rotation/translation	true
AutomaticTransformInitialization	Initial allignment	true
NumberOfResolutions	Factor used to smooth images	4
MaximumNumberOfIterations	Iterations used for computation	2000
NumberOfHistogramBins	Size of histogram	32
ImageSampler	Fixed image sampler	RandomCoordinate
BSplineInterpolationOrder	Order of B-spline polynomial	3
FinalBSplineInterpolationOrder	Order of B-spline used to resample moving image	3

**Elastix Parameters (nonrigid)**		
Parameter	Description	Value
Registration	Class of registration framework	MultiResolutionRegistration
FixedImagePyramid	Morphological scale space	FixedSmoothingImagePyramid
MovingImagePyramid	Morphological scale space	MovingSmoothingImagePyramid
Interpolator	Evaluation of voxel intensity	BSplineInterpolator

Metric	Similarity measure	AdvancedNormalizedCorrelation
Optimizer	Optimal transformation parameter	AdaptiveStochasticGradientDescent
AGSDParameterEstimationMethod	Method for estimating the parameters for Adaptive Stochastic Gradient Descent	DisplacementDistribution
AutomaticTransformInitialization	Automatically guess an initial translation by aligning the geometric centers of the fixed and moving	true
AutomaticScalesEstimation	Method to estimate scales	true
NumberOfSpatialSamples	Samples used to compute similiarity	3750
NewSamplesEveryIteration	Whether to refresh the spatial samples	true
ResampleInterpolator	Intensity interpolation during resampling	FinalBSplineInterpolator
Resampler	Calculation of deformed image	DefaultResampler
Transform	Deformation used	BSplineTransform
AutomaticScalesEstimation	Scales between rotation/translation	true
AutomaticTransformInitialization	Initial allignment	true
NumberOfResolutions	Factor used to smooth images	4
MaximumNumberOfIterations	Iterations used for computation	2000
NumberOfHistogramBins	Size of histogram	32
ImageSampler	Fixed image sampler	RandomCoordinate
BSplineInterpolationOrder	Order of B-spline polynomial	3
FinalBSplineInterpolationOrder	Order of B-spline used to resample moving image	3

FinalGridSpacingInVoxels	B-spline control point spacing for deformation	8

Niftyreg parameters (nonrigid)		
Parameter	Description	Value
be_val	Bending energy penalty weight	0.005
le_val	Linear elasticity penalty weight	0.000
sx_val	Control point grid spacing x	0.4
sy_val	Control point grid spacing y	0.4
ln_val	Number of resolution levels	4
maxit_val	Maximum iterations per level	300
nmi_flag	Use normalized mutual information	True
vel_flag	Use velocity field integration	True
pad_val	Image padding value	0

Niftyreg parameters (rigid)		
Parameter	Description	Value
pv_val	Percentage of blocks considered for registration	70
pi_val	Percentage of inlier blocks used for optimization	70
ln_val	Number of resolution levels	3
maxit_val	Maximum iterations per level	10
smoo_val	Smoothing factor	0.5
rigOnly	Restricts to rigid transformation only	True
lp	Pyramid levels for moving image	3

ANTs parameters (nonrigid)		
Parameter	Description	Value
reg_iterations	Registration iterations per level	(80,40,10)
grad_step	Gradient step size	0.2
flow_sigma	Flow field smoothing	4

total_sigma	Total field smoothing	0
syn_sampling	Sampling percentage	32
type_of_transform	Transformation type	SyNOnly

**ANTs parameters (rigid)**		
Parameter	Description	Value
aff_iterations	Registration iterations per level	(2100,1200,1200,10)
aff_shrink_factors	Multi-resolution shrinking factors	(4,2,1,1)
aff_smoothing_sigmas	Gaussian smoothing per level	(4,3,2,0)
aff_random_sampling_rate	Rate of random sampling	0.3
aff_metric	Similarity metric	Mattes
aff_sampling	Percentage of data sampled	32
type_of_transform	Transformation type	Rigid

**FILRT parameters**		
Parameter	Description	Value
Bins	Number of histogram bins	32
interp	Interpolation method	Trilinear
searchrx	Search range in x direction	10
searchry	Search range in y direction	10
dof	Degrees of freedom	3
searchcost	Cost function for search	Corratio
cost	Similarity metric	normcorr
